# The low expression of miR-155 promotes the expression of SHP2 by inhibiting the activation of the ERK1/2 pathway and improves cell pyroptosis induced by I/R in mice

**DOI:** 10.18632/aging.205631

**Published:** 2024-03-06

**Authors:** Mengru Liu, Dongliang Fu, Tong Gao, Hong Jiang, Peng Yang, Xianlun Li

**Affiliations:** 1 Department of Integrative Medicine Cardiology, China-Japan Friendship Hospital, Beijing 100029, China; 2 Department of Cardiology, Beijing Tsinghua Changgung Hospital, Beijing 100029, China

**Keywords:** miR-155, SHP2, I/R, pyroptosis

## Abstract

This study aims to explore the specific mechanism by which miR-155 regulates SHP2 expression in mouse ischemia-reperfusion (I/R) induced necroptosis. Various methods including cardiac ultrasound, TTC staining, Masson staining, TUNEL staining, and Western blotting were used to examine changes in the morphology and function of the rat left ventricle, myocardial fibrosis, as well as the expression of proteins related to tissue and cardiomyocyte necroptosis pathways. *In vivo* results showed that knockdown (KD) of miR-155 significantly improved cardiac ultrasound parameters (EF, FS, LVAW;d, and LVAW;s), reduced the myocardial infarction area, myocardial fibrosis, and cell apoptosis in I/R mice, upregulated cardiac SHP2 protein expression, and other proteins including p-ERK1/2, NLRP3, GSDMD, caspase-3, caspase-4, and caspase-11 were also significantly decreased. *In vitro* experiments showed that compared with the SHP2 WT miR-155 KD group, SHP2 protein expression was significantly increased in the SHP2 WT miR-155 KD group, while the expression of other proteins was significantly reduced, consistent with *in vivo* results. MiR-155 can regulate ERK1/2 and NLRP3 through SHP2. After adding the ERK1/2 inhibitor U0126 to cardiomyocytes from SHP2 KO mice, it was found that the expression of proteins other than SHP2 significantly decreased compared to SHP2 KO cells without the inhibitor. In summary, low expression of miR-155 promoted the expression of SHP2 and improved mouse I/R-induced necroptosis by inhibiting the activation of the ERK1/2 pathway.

## INTRODUCTION

Myocardial ischemia/reperfusion injury (MIRI) refers to the phenomenon where myocardial injury is more severe after the restoration of coronary artery blood flow for a certain period than during ischemia due to coronary artery disease, preventing blood from passing through the coronary arteries [1]. Reperfusion can cause arrhythmias and microcirculation disorders, leading to an increase in the area of myocardial infarction and the extent of myocardial damage. Previous strategies to reduce myocardial apoptosis, such as scavenging free radicals, relieving calcium overload, anti-inflammatory measures, and regulating autophagy, have not shown significant therapeutic effects in clinical treatments [2, 3]. Considering the bottlenecks in the prevention and treatment of MIRI, there is now recognition that other forms of myocardial injury may exist.

In recent years, the role of necroptosis in cardiovascular diseases has garnered widespread attention. Necroptosis, characterized by rapid rupture of the cell membrane and release of inflammatory intracellular contents, is a unique form of programmed cell death that is distinct from apoptosis and necrosis [4]. Caspase-1 plays a decisive role in the formation of necroptosis and can lead to a cascade of tissue damage [5]. Studies have found that after activation of the NLR family pyrin domain containing 3 (NLRP3), precursor caspase-1 is converted into cleaved caspase-1 by adaptor proteins, and then cleaved caspase-1 induces the maturation of interleukin-1β (IL-1β), leading to necroptosis [6]. Moreover, myocardial NLRP3-mediated necroptosis has been confirmed to be an important factor in the occurrence of MIRI, and MIRI is associated with the NLRP3 inflammasome [7]. Therefore, effectively inhibiting the activation of the NLRP3 inflammasome can reduce necroptosis, thereby alleviating MIRI.

Previous literature reports that micR-155 can participate in focal cerebral ischemia models [8]. Src homology 2 (SH2) domain-containing protein tyrosine phosphatase-2 (SHP2) has been proven to play an important role in cell proliferation and differentiation in growth factors and cytokine signaling (such as the Ras-Raf-ERK cascade induced by epidermal growth factor and platelet-derived growth factor) [9, 10]. SHP2 may prevent necroptosis by directly binding to JNK and inhibiting its phosphorylation [11]. For instance, the ablation or inhibition of SHP2 in cardiomyocytes enhances the activation of NLRP3, promotes the overproduction of pro-inflammatory cytokines interleukin-1β (IL-1β) and IL-18, and increases sensitivity to peritonitis12, indicating that SHP2 is a negative regulator of the NLRP3 inflammasome. Inhibiting SHP2 can enhance the sensitivity of tumors to PD-1 therapy, thus enhancing the pro-apoptotic effect12. In recent years, new evidence has emerged suggesting that miRNAs regulate the onset and progression of diseases through targeting SHP2.

In the present study, therefore, the mouse model of MIRI was established using cardiomyocyte-specific conditional SHP2-knockout (cSHP2-KO) mice, and the specific mechanism of miR-155 regulating the expression of SHP2 in mouse I/R-induced pyroptosis was explored.

## MATERIALS AND METHODS

### Bioinformatics analysis

The datasets related to MI were searched for from the Gene Expression Omnibus (GEO) database (https://www.ncbi.nlm.nih.gov/gds/). The dataset GSE24591 of MI-related mRNA expression and the dataset GSE76591 containing miRNA sequencing of MI were downloaded. The non-coding RNA profiling by array data was subjected to quantile normalization using the limma package of R software, and the differentially expressed genes (DEGs) were analyzed (|logFC|<1, *p* < 0.05). In particular, for the expression profile dataset we obtained, the data are first log2-transformed. We then apply the limit function to perform multiple linear regression. Further, we utilize the eBays function to compute moderated t-statistics, moderated F-statistics, and log-odds of differential expression by empirical Bayes moderation of the standard errors towards a common value, ultimately obtaining the significance of differential expression for each gene, and the cluster analysis heat map of DEGs was plotted using the heatmap package of R software. Similarly, the volcano plot of visual grouping of DEGs in the dataset GSE76591, and the cluster analysis heat map of DEGs were plotted.

### Functional enrichment analysis

The DEGs in GSE24591 were subjected to Gene Ontology (GO) and Kyoto Encyclopedia of Genes and Genomes (KEGG) enrichment analyses. The DEGs at the biological process, cellular component, and molecular function levels were analyzed using the online database tool DAVID (https://david.ncifcrf.gov) to integrate the GO terms, and the biological process network of DEGs was also created. The GO and KEGG pathway enrichment analysis diagrams of DEGs were plotted using the GOplot and ggplot2 packages of R software.

### Gene set enrichment analysis (GSEA)

The GSEA (Gene Set Enrichment Analysis) software (version 3.0) was obtained from the website. Samples were divided into two groups based on grouping information, and a subset of the Molecular Signatures Database (https://doi.org/10.1093/bioinformatics/btr260, http://www.gsea-msigdb.org/gsea/downloads.jsp) named c2.cp.v7.4.symbols.gmt was downloaded for assessing relevant pathways and molecular mechanisms. The analysis was performed based on gene expression profiles and phenotype grouping, with a minimum gene set size of 5 and a maximum gene set size of 5000. A total of one thousand resamplings were conducted and results with a *P*-value of <0.05 (as needed) and a False Discovery Rate (FDR) of <0.25 (as needed) were considered statistically significant.

### Protein-protein interaction (PPI) network analysis of DEGs and target gene screening

The DEGs were entered into the online tool STRING to screen the interacting protein with a combined score >0.9. Then the PPI results obtained were imported into Cytoscape software, and the target genes with a score <10 were obtained using the degree algorithm.

### Prediction of miRNA target genes

The candidate miRNA target genes were predicted using the online tools starBase and TargetScan, and using the multiMiR package, filter genes within the top 90^th^ percentile, search for miRNA binding sites associated with PTPN11, and create an UpSet plot. Moreover, the binding sites between mRNA and miRNA were plotted based on the prediction results.

### Animal modeling and grouping

The MIRI model was established according to the method in the literature [7]. After the mice were anesthetized with an intraperitoneal injection of pentobarbital sodium (50 mg/kg) and received ventilator support, the skin in the operating area was prepared and disinfected with iodine and alcohol. An about 3 cm-long skin incision was made transversely between the left fourth and fifth intercostal spaces in the thoracic area, the first- and second-layer intercostal muscles were cut and slightly separated, the third-layer intercostal muscle was bluntly separated, the heart was exposed with a chest opener, and the pericardium was separated, followed by ligation (slipknot) of the left anterior descending coronary artery (a small pad was placed between the ligatures to prevent myocardial laceration) 2–3 mm below the left atrial appendage. After that, the left ventricular anterior wall tissue turned from red to yellow-white indicating successful ligation. Then the chest cavity was closed. 30 min later, the chest cavity was reopened, the ligature was cut off with scissors, the chest cavity was closed and the skin was sutured (the tissue specimens were harvested after 24 h). All animals were randomly divided into sham group, model group, and miR-155 KD group. In the sham group, the mice were treated only with operation, without coronary artery ligation. In the model group, I/R was performed. In the miR-155 KD group, the mice were injected with miR-155 inhibitor via the caudal vein. The optimal dose and time show miR-155 inhibitor was 20 mg/kg in 0.2 ml per injection after 6 h of molding.

### Cardiac ultrasonography

After inhalation anesthesia with 2.0% isoflurane, the mice were fixed in a supine position on a 37°C constant temperature heating plate, and the chest and upper abdomen were depilated to fully expose the skin, followed by ultrasonography using a Vevo 2100 small animal ultrasound machine with an MS-550D probe. On the sternal long-axis view, the probe was rotated 90° clockwise based on the left ventricular long-axis view, *i.e*., the left ventricular short-axis view, and the left ventricular motion status was recorded in an M-mode: left ventricular ejection fraction (EF), left ventricular fractional shortening (FS), left ventricular end-diastolic anterior wall thickness (LVAW; d) and left ventricular end-systolic anterior wall thickness (LVAW; s).

### Culture and processing of cardiomyocyte

After I/R, cSHP2-KO and wild-type (WT) mice were anesthetized with 2% isoflurane and sacrificed by cervical dislocation, from which the bone marrow cardiomyocytes were separated, with six mice in each group. The tibia and femur of mice aged 6–8 weeks were separated, and cells were washed with pre-cooled phosphate buffer saline (PBS)+2% fetal bovine serum (FBS) until they turned white. Then the resulting bone marrow mixture was filtered through a filter mesh to obtain the bone marrow single-cell suspension, followed by centrifugation at 1,000 rpm and 4°C. The cells were transfected with 20 nmol/L NC mimic and 20 nmol/L miR-155 mimic, respectively, with Lipofectamine 3000 reagent, while the ERK inhibitor U0126 was added into the medium of cardiomyocytes derived from KO mice. In this way, the cells were divided into six groups: SHP2 WT NC group, SHP2 WT miR-155 KD group, SHP2 KO NC group, SHP2 KO miR-155 KD group, SHP2 KO NC + U0126 group, and SHP2 KO miR-155 KD+ U0126 group.

### Detection of MI area by 2,3,5-triphenyl tetrazolium chloride (TTC) staining

The myocardial tissues were quickly frozen in a refrigerator at −20°C for 10 min, sliced into 5 sections of about 2 mm in thickness, and incubated in 2% TTC PBS away from light by tin foil paper at 37°C for 30 min, followed by staining on the other side. Normal tissues were stained red, but the infarction region was not stained. Then the sections were fixed with 4% paraformaldehyde for 12 h and photographed, and the photos were analyzed using ImageJ software. Finally, the MI area was calculated.

### Masson staining of myocardial tissues

After deparaffinization, the paraffin sections were immersed in mordant, and dried in a constant temperature incubator at 60°C for 1 h. After washing, the sections were instilled with celestine blue and Mayer’s Hematoxylin, differentiated with acidic ethanol, instilled with Ponceau S, washed with water, and added with phosphomolybdic acid hydrate. After the liquid was poured away, the sections were added with aniline blue, washed with weak acid, dehydrated with 95% ethanol and absolute ethanol, naturally dried, mounted with neutral balsam, and covered with a cover glass. The changes in collagen fibers in myocardial tissues were observed and photographed under a microscope. Finally, the myocardial fibrosis (MF) region was measured using Image Pro-Plus-6 software (Media Cybernetics).

### Detection of apoptosis by terminal deoxynucleotidyl transferase-mediated dUTP nick end labeling (TUNEL) staining

By the instructions of TUNEL kits, the samples were permeabilized with 0.1% TritonX-100, added with 200 μL of 3% H_2_O_2,_ and washed with PBS 3 times, and both DNA and RNA were inactivated by proteinase K, followed by incubation in an incubator away from light for 60 min. Then the samples were washed with PBS 3 times, reacted with DAPI solution for 20 min, washed, and mounted. The apoptosis in the left ventricular tissue sections was observed under a microscope. Apoptosis index (AI) = number of positive cells in each field/total cells in each field × 100%.

### Western blotting

An appropriate number of cryopreserved heart tissues and cells were collected, lysed in an ice bath, and homogenized. The supernatant was extracted, and the total protein concentration of cells and tissues was measured by the BCA method. After balancing, the protein was subjected to SDS-PAGE, transferred onto a membrane, sealed with 5% skim milk powder for 2 h, and incubated with primary antibodies (SHP2, p-ERK1/2, t-ERK1/2, NLRP3, GSDMD, caspase-1, caspase-3, caspase-4, caspase-11 and GAPDH) on a horizontal shaker at 4°C overnight. After the membrane was washed, the protein was incubated again with secondary antibodies (1:5,000) on the horizontal shaker at room temperature for 2 h, followed by ECL color development and photography using the gel imaging system. The assay was repeated 3 times. The relative protein expression was expressed as the ratio of the gray value in each group to that of the internal reference detected by ImageJ.

### Statistical analysis

The DEseq2 and ggpubr packages of R software (v3.6.1) were used for bioinformatics analysis. DEGs were analyzed by the Wald test, and cytokines were compared between two groups by rank sum test. SPSS 23.0 software was used for statistical analysis of other indexes. Measurement data were expressed as mean ± standard deviation (*x̄* ± s). A one-way analysis of variance was adopted for comparison among groups, and an LSD test for pairwise comparison. *P* < 0.05 was considered to be statistically significant.

### Availability of data and materials

The datasets used and analyzed during the current study are available from the corresponding author upon reasonable request.

## RESULTS

### Bioinformatics analysis identified the upregulated gene microRNA-155 during ischemia-reperfusion

**Figure 1:**
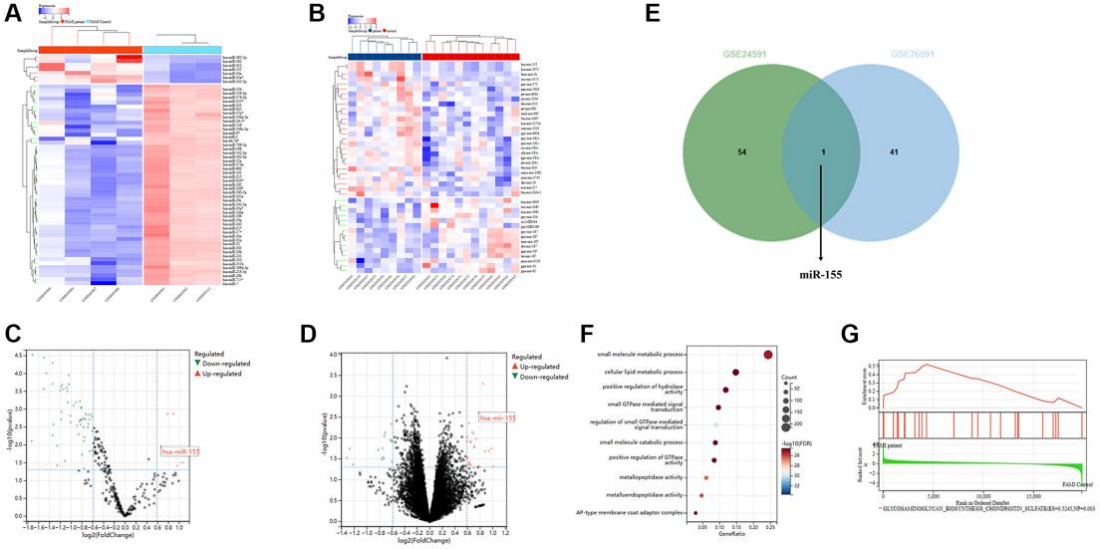
**Bioinformatics analysis results.** (**A**, **B**) Heatmaps of clustered differentially expressed genes (DEGs) in GSE24591 and GSE76591. (**C**, **D**) Volcano plots of DEGs in GSE24591 and GSE76591. (**E**) Venn diagram. (**F**) KEGG pathways. (**G**) GSEA.

Data were downloaded from the GEO database, including GSE24591 and GSE76591. Subsequent analyses were then conducted on GSE24591 with the criteria (*p* < 0.05 and |logFC| < 1). GSE24591 is composed of 15,569 genes and 284 miRNA entries, while GSE76591 consists of 21 gene entries with a total of 19,832 genes. In the miRNA differential analysis for GSE24591, 7 genes were found to be upregulated and 55 genes were downregulated (Figure 1A, 1B). We created volcano plots for GSE24591 and GSE76591 (Figure 1C, 1D). By taking the intersection of the microRNAs from GSE24591 and GSE76591, microRNA-155 was identified as the sole major molecule in the ischemia-reperfusion model under investigation (Figure 1E). Moreover, KEGG enrichment analysis of DEGs was executed and KEGG pathway maps were plotted (Figure 1F). GSEA (Gene Set Enrichment Analysis) revealed enriched pathways (Figure 1G).

### Changes in morphological structure and function of the left ventricle in mice

**Figure 2:**
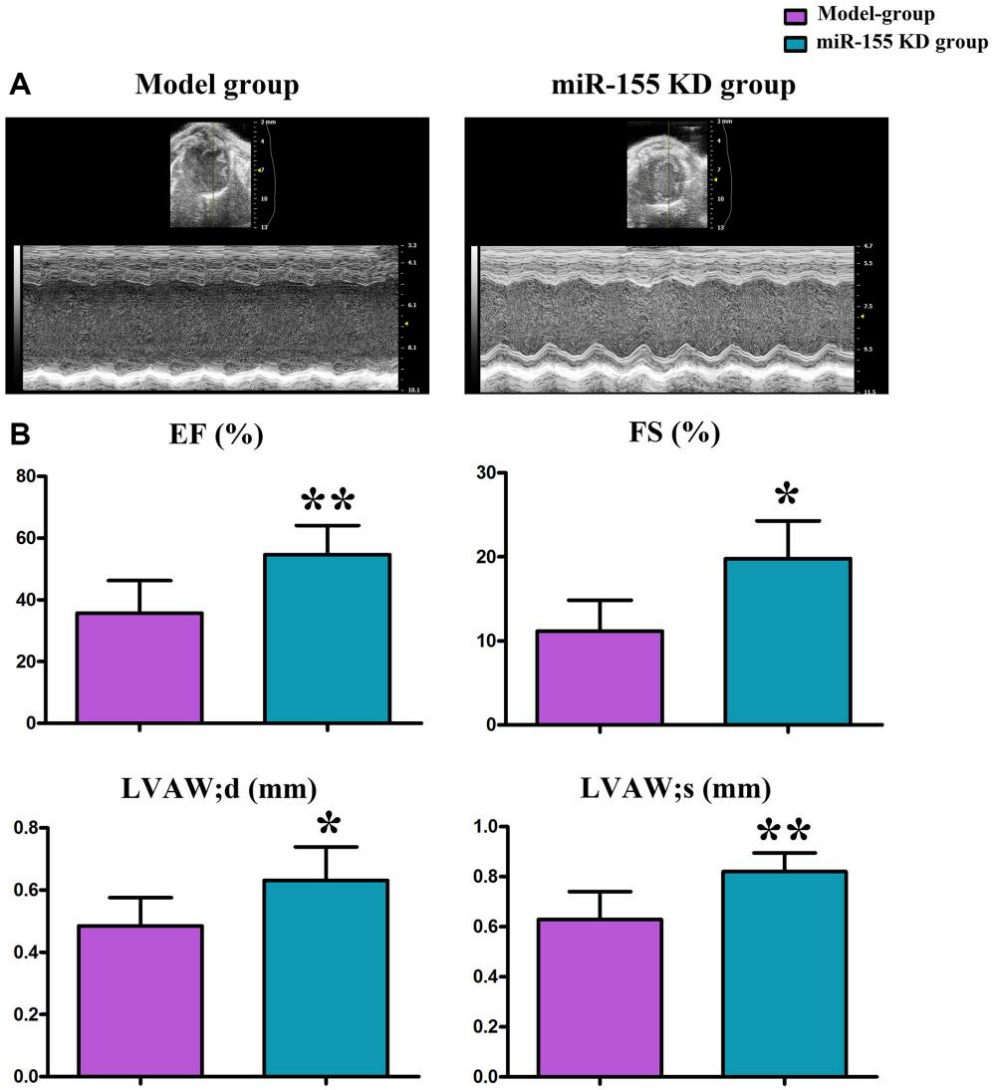
**Changes in left ventricular morphology and function as shown by cardiac ultrasound.** (**A**) Cardiac ultrasound. (**B**) Changes in EF (%), FS (%), LVAWd (mm), and LVAW; (mm).

Ultrasound examination revealed significant increases in EF (%), FS (%), LVAW’d (mm), and LVAW’s (mm) in the miR-155 KD group compared to the model group. (Figure 2).

### Changes in MF in mice

**Figure 3:**
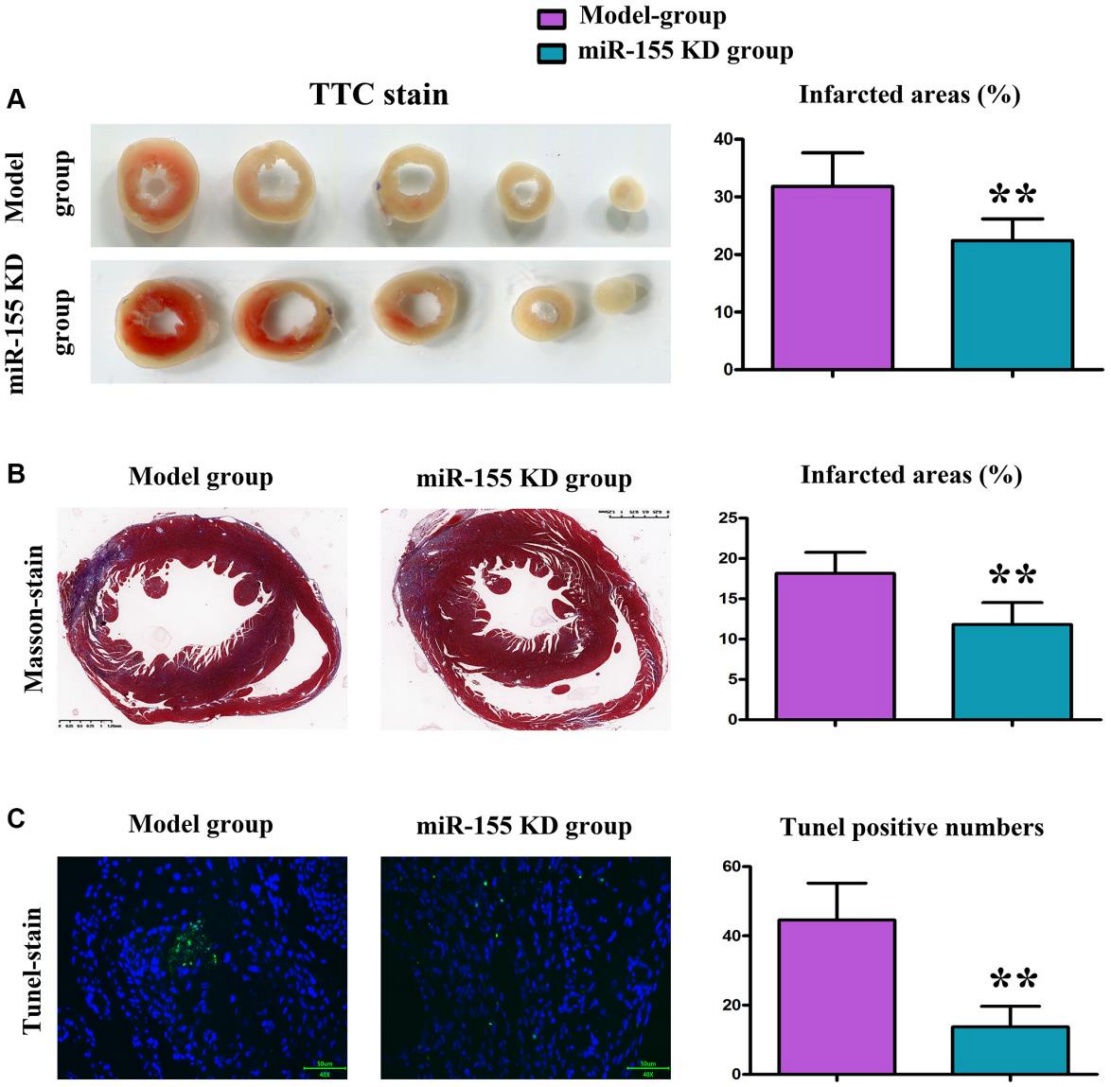
**Changes in myocardial infarction (MI) in mice.** (**A**) TTC staining of myocardial tissue and MI area. (**B**) Masson staining of myocardial tissue and MI area. (**C**) Results of TUNEL staining and number of positive apoptotic cells.

Gross observation showed that rats in the model group displayed evident MI (myocardial infarction) and MF (myelofibrosis); pathologically, TTC (triphenyl tetrazolium chloride) and Masson’s trichrome staining results revealed that miR-155 knockdown (KD) significantly reduced the area of MI in the myocardium of mice from the model group after injury and alleviated MF. (Figure 3A, 3B).

### Low-expression of miR-155 ameliorated apoptosis of heart tissues

TUNEL staining showed apoptotic cells in green and total cells in blue. The number of positive cells in the cardiac tissues of miR-155 KD group mice was significantly reduced compared to the model group, suggesting that low expression of miR-155 can improve apoptosis in the cardiac tissue of mice in the model group. (Figure 3C).

### Prediction of miRNA target genes

**Figure 4:**
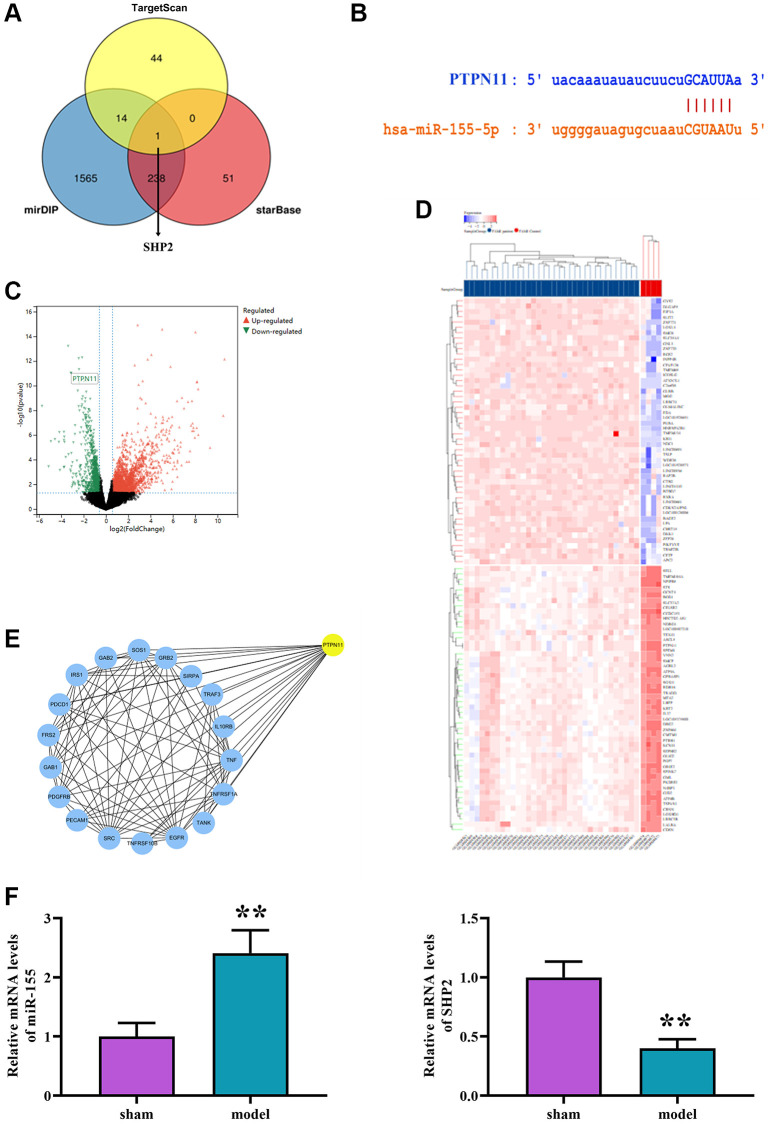
(**A**) Intersection prediction of downstream genes of miR-155 as SHP2 using TargetScan, mirDIP, and starBase. (**B**) Binding sites between mRNA and miRNA. (**C**) Volcano plot of DEGs in GSE24591. (**D**) Clustering heatmap of DEGs in GSE24591. (**E**) PPI network. (**F**) The levels of micRNA-155 expression and SHP2 mRNA were measured.

Online tools such as starBase and TargetScan were utilized to predict candidate miRNA target genes, revealing overlapping target genes bound by miRNAs. Results indicated that miR-155 consistently exists across three databases. (Figure 4A) Based on prediction results, the binding sites between mRNA and miRNA were illustrated, with SHP2 identified as a key downstream gene of miR-155 and a predicted binding site (Figure 4B). GSE24591 volcano plot analysis showed significant downregulation of SHP2 (Figure 4C), and GSE24591 analysis revealed Fifty up-regulated genes (Figure 4D). DEGs were imported into the STRING database to obtain a PPI network. The PPI network was then imported into Cytoscape software, and the cytoHubba plugin was used to obtain target genes with scores of <10 (Figure 4E). Compared to the sham group, the model group exhibited an increase in the expression of micRNA 155 and a decrease in the expression of SHP2 (Figure 4F).

### Low-expression of miR-155 ameliorated NLRP3-induced pyroptosis after I/R in mice

**Figure 5:**
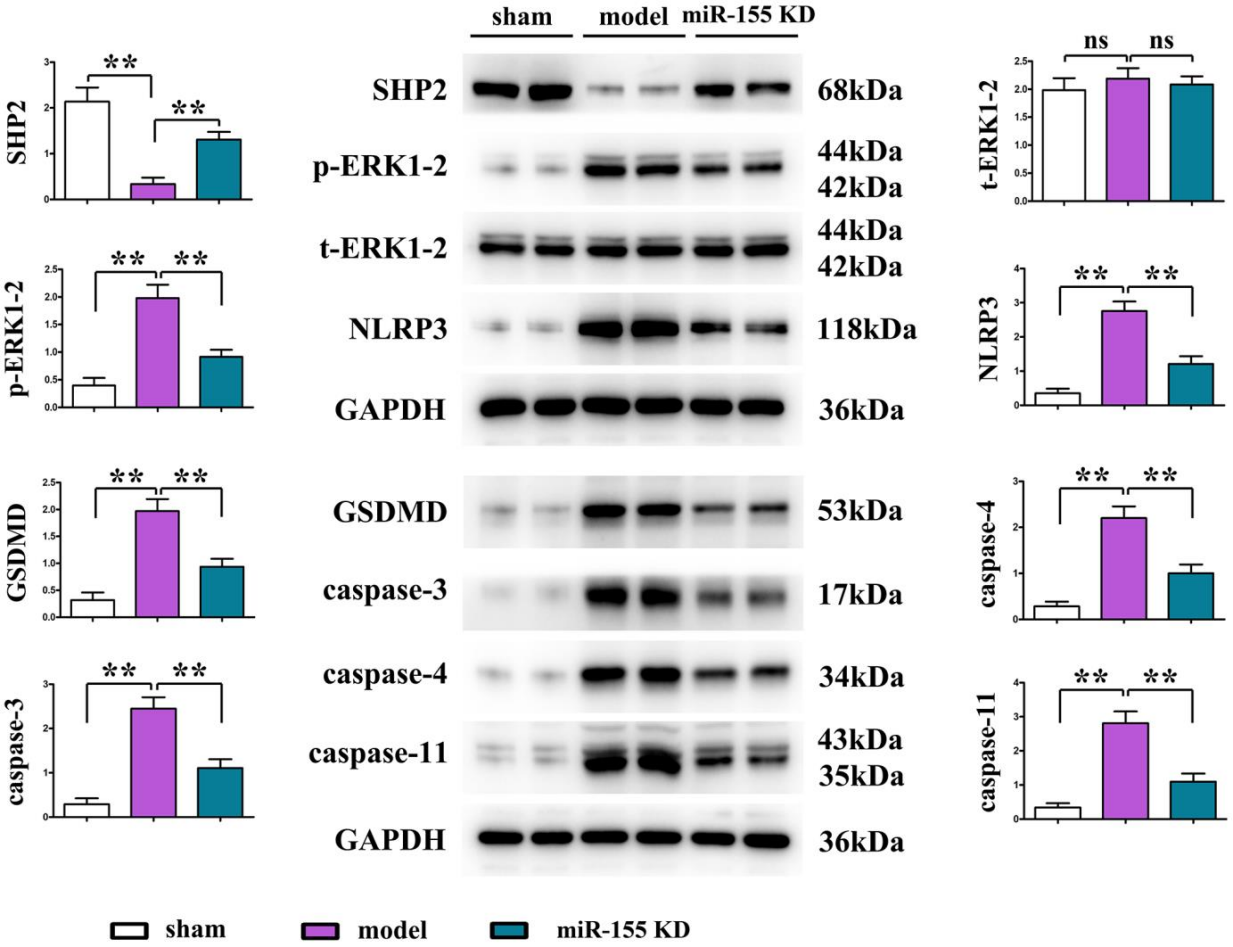
Protein expression of SHP2, p-ERK1/2, t-ERK1/2, NLRP3, GSDMD, caspase-3, caspase-4, caspase-11 in cardiac tissue.

Compared to the sham surgery group, the cardiac tissue of rats in the model group exhibited a significant decrease in SHP2 protein expression, while the expression of p-ERK1/2, NLRP3, GSDMD, caspase-3, caspase-4, and caspase-11 proteins was significantly increased. Low expression of miR-155 significantly upregulated the protein expression of SHP2 in the myocardium of I/R mice, which in turn significantly downregulated the protein expression of p-ERK1/2, NLRP3, GSDMD, caspase-3, caspase-4, and caspase-11. It can be seen that the expression of miR-155 may reduce pyroptosis in I/R mice by upregulating the protein expression of SHP2 and inhibiting the activation of p-ERK1/2 and NLRP3. (Figure 5).

### Low-expression of miR-155 promoted expression of SHP2 and ameliorated mouse I/R-induced pyroptosis through inhibiting activation of ERK1/2 pathway

**Figure 6:**
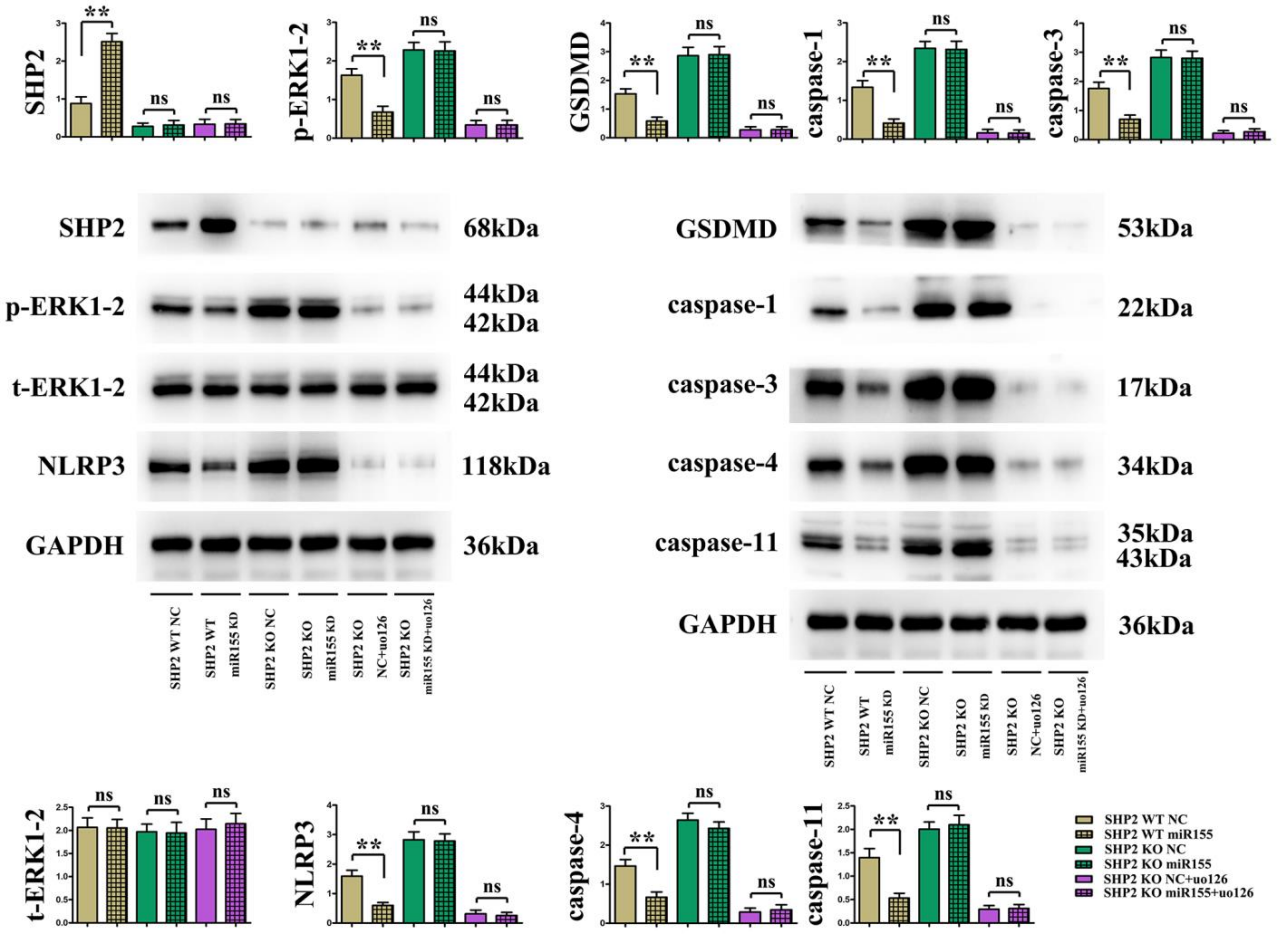
Protein expression of SHP2, p-ERK1/2, t-ERK1/2, NLRP3, GSDMD, caspase-1, caspase-3, caspase-4, caspase-11 in cardiomyocytes.

In cardiomyocytes derived from WT mice, compared to the SHP2 WT NC (negative control) group, the SHP2 protein expression was significantly increased in the SHP2 WT miR-155 KD (knockdown) group, while the expression of other proteins such as p-ERK1/2, NLRP3, GSDMD, caspase-3, caspase-4, and caspase-11 was significantly decreased, consistent with *in vitro* experimental results. Compared to the SHP2 KO (knockout) NC group, there was no significant change in the expression of the aforementioned proteins in the myocardial cells of SHP2 KO miR-155 KD group mice. The absence of SHP2 negates the regulatory effects of miR-155 on ERK1/2 and NLRP3, suggesting that the low expression of miR-155 affects the expression of proteins related to pyroptosis in I/R mice by promoting the expression of SHP2. Moreover, after adding the ERK1/2 inhibitor U0126 to myocardial cells from KO mice, the expression of p-ERK1/2, NLRP3, GSDMD, caspase-3, caspase-4, and caspase-11 was significantly decreased compared to SHP2 KO cells without the inhibitor. This suggests that NLRP3-induced apoptosis in myocardial cells of I/R mice is regulated by the ERK signaling pathway (Figure 6).

## DISCUSSION

As a prevalent disease globally, cardiovascular diseases have a high mortality rate [12]. Currently, there have been more than 290 million patients with cardiovascular diseases, ranking 1^st^ among diseases, and cardiovascular mortality is always on the rise [13]. IRI has long been considered one of the factors causing adverse cardiovascular outcomes after myocardial ischemia, cardiac surgery, or circulatory arrest, which usually occurs following I/R in patients with MI, coronary heart disease, and stroke [14]. Therefore, the way to relieve IRI is particularly important for heart disease. In the present study, the bioinformatics analysis showed that miR-155 had a low expression in the heart tissues of I/R mice, suggesting that miR-155 may act as an important player in myocardial injury in I/R mice. Therefore, the mouse model of MIRI was established to study the effect of miR-155 on I/R in this experiment. It was found that expression of miR-155 significantly reduced the MI area and MF after myocardial injury in mice. Then the mechanism of low-expression of miR-155 protecting against MIRI in mice was further explored.

In recent years, the role of pyroptosis in cardiovascular diseases has attracted great attention. Pyroptosis, a form of caspase-1-dependent cell death, refers to the suicidal behavior from a series of regulatory responses to hypoxic stimulus. Inflammatory necrosis is usually mediated by GSDMD, and it is caspase-1 in the NLRP3 inflammasome pathway that activates GSDMD [15]. Therefore, pyroptosis occurs actually through the NLRP3 inflammasome, displaying a close connection between the two. Pyroptosis plays a vital role in inflammation, which has been widely studied in different cardiovascular diseases [16]. It has been confirmed that pyroptosis is implicated in cardiomyocyte hypoxia/reoxygenation injury and cerebral IRI [17]. In addition, according to a large number of studies, inhibiting pyroptosis can effectively alleviate MIRI. For example, emodin relieves MIRI-induced pyroptosis via inhibiting GSDMD [18], and miR-29a alleviates I/R by targeting SIRT1 and suppressing oxidative stress and the NLRP3-mediated apoptosis pathway [19]. In the present study, low-expression of miR-155 significantly up-regulated the protein expression of SHP2 in the myocardium in I/R mice but inhibited the protein expression of p-ERK1/2, while the expression of pyroptosis-related proteins NLRP3, GSDMD, caspase-3, caspase-4, and caspase-11 also significantly declined. The above findings suggest that the protective effect of the low expression of miR-155 against I/R in mice may be related to the pyroptosis signaling pathway, and low expression of miR-155 may inhibit the activation of p-ERK1/2 and NLRP3 by up-regulating the protein expression of SHP2, thus ameliorating I/R-induced pyroptosis in mice.

The specific mechanism by which low expression of miR-155 improves cell pyroptosis induced by I/R in mice was further verified through cellular experiments. Results indicated that, compared to the SHP2 WT NC group, the expression of the SHP2 protein was significantly increased in the SHP2 WT miR-155 KD group, while the expression of other proteins such as p-ERK1/2, NLRP3, GSDMD, caspase-3, caspase-4, and caspase-11 was significantly decreased, consistent with *in vivo* experimental results. This further suggests that the ability of low expressed miR-155 to alleviate cell pyroptosis induced by I/R in mice may be related to the expression of SHP2 protein and the activation of p-ERK1/2. Compared with SHP2 WT mice, the expression of these proteins was upregulated in SHP2 KO mice, indicating that SHP2 has a certain inhibitory effect on the activation of p-ERK. Moreover, there was no significant change in the expression of these proteins in the SHP2 KO miR-155 KD group and SHP2 KO NC group cardiomyocytes, indicating that the absence of SHP2 deprives the regulatory effect of low expressed miR-155 on ERK1/2 and NLRP3. This suggests that low expressed miR-155 affects the expression of I/R-induced pyroptosis-related proteins in mice by promoting the expression of SHP2. Moreover, after adding the ERK1/2 inhibitor U0126 to cardiomyocytes in KO mice, the expression of p-ERK1/2, NLRP3, GSDMD, caspase-3, caspase-4, and caspase-11 was significantly reduced compared to SHP2 KO cells without the inhibitor, suggesting that the apoptosis of I/R-induced cells in mice induced by nlrp3 is regulated by p-ERK1/2. In other words, SHP2 inhibits the activation of p-ERK, thereby reducing cell pyroptosis induced by nlrp3 after I/R.

## References

[1] Wu Y, Liu H, Wang X. Cardioprotection of pharmacological postconditioning on myocardial ischemia/reperfusion injury. Life Sci. 2021; 264:118628. 10.1016/j.lfs.2020.118628 33131670

[2] Bernink FJ, Timmers L, Beek AM, Diamant M, Roos ST, Van Rossum AC, Appelman Y. Progression in attenuating myocardial reperfusion injury: an overview. Int J Cardiol. 2014; 170:261–9. 10.1016/j.ijcard.2013.11.007 24289874

[3] Ibanez B, Fuster V, Jiménez-Borreguero J, Badimon JJ. Lethal myocardial reperfusion injury: a necessary evil? Int J Cardiol. 2011; 151:3–11. 10.1016/j.ijcard.2010.10.056 21093938

[4] Aachoui Y, Sagulenko V, Miao EA, Stacey KJ. Inflammasome-mediated pyroptotic and apoptotic cell death, and defense against infection. Curr Opin Microbiol. 2013; 16:319–26. 10.1016/j.mib.2013.04.004 23707339 PMC3742712

[5] Miao EA, Rajan JV, Aderem A. Caspase-1-induced pyroptotic cell death. Immunol Rev. 2011; 243:206–14. 10.1111/j.1600-065X.2011.01044.x 21884178 PMC3609431

[6] Rathinam VA, Fitzgerald KA. Inflammasome Complexes: Emerging Mechanisms and Effector Functions. Cell. 2016; 165:792–800. 10.1016/j.cell.2016.03.046 27153493 PMC5503689

[7] Nie C, Ding X, A R, Zheng M, Li Z, Pan S, Yang W. Hydrogen gas inhalation alleviates myocardial ischemia-reperfusion injury by the inhibition of oxidative stress and NLRP3-mediated pyroptosis in rats. Life Sci. 2021; 272:119248. 10.1016/j.lfs.2021.119248 33621592

[8] Silva JPD, Lizarte Neto FS, Cirino MLA, Carvalho CAM, Carlotti CG Jr, Colli BO, Tirapelli DPDC, Tirapelli LF. Analysis of Caspase-9 protein and microRNAs miR-21, miR-126 and miR-155 related to the apoptosis mechanism in the cerebellum of rats submitted to focal cerebral ischemia associated with an alcoholism model. Arq Neuropsiquiatr. 2019; 77:689–95. 10.1590/0004-282X20190126 31664344

[9] Agazie YM, Hayman MJ. Molecular mechanism for a role of SHP2 in epidermal growth factor receptor signaling. Mol Cell Biol. 2003; 23:7875–86. 10.1128/MCB.23.21.7875-7886.2003 14560030 PMC207628

[10] De R. Noonan syndrome causing-SHP2 mutants inhibit IGF-I release via GH-induced ERK1/2 hyper-activation, which contributes to short stature. Annales Dendocrinologie. 2012; 73:251–2.

[11] Chen C, Cheng Y, Lei H, Feng X, Zhang H, Qi L, Wan J, Xu H, Zhao X, Zhang Y, Yang B. SHP2 potentiates anti-PD-1 effectiveness through intervening cell pyroptosis resistance in triple-negative breast cancer. Biomed Pharmacother. 2023; 168:115797. 10.1016/j.biopha.2023.115797 37913735

[12] Li L, Li X, Zhang Z, Liu L, Zhou Y, Liu F. Protective Mechanism and Clinical Application of Hydrogen in Myocardial Ischemia-reperfusion Injury. Pak J Biol Sci. 2020; 23:103–12. 10.3923/pjbs.2020.103.112 31944068

[13] Wang P, Zhang B, Jin L, Liao H, Dong T. Association of various risk factors with prognosis and hospitalization cost in Chinese patients with acute myocardial infarction: A clinical analysis of 627 cases. Exp Ther Med. 2015; 9:603–11. 10.3892/etm.2014.2087 25574242 PMC4280932

[14] Omoto ACM, Gava FN, Silva CAA, Silva HB, Parente JM, Costa RM, Castro MM, Tostes RC, Salgado HC, Fazan R Jr. Lack of scarring is not always a sign of cardiac health: Functional and molecular characterization of the rat heart's following chronic reperfusion. PLoS One. 2018; 13:e0209190. 10.1371/journal.pone.0209190 30571725 PMC6301775

[15] Jia C, Chen H, Zhang J, Zhou K, Zhuge Y, Niu C, Qiu J, Rong X, Shi Z, Xiao J, Shi Y, Chu M. Role of pyroptosis in cardiovascular diseases. Int Immunopharmacol. 2019; 67:311–8. 10.1016/j.intimp.2018.12.028 30572256

[16] Del Re DP, Amgalan D, Linkermann A, Liu Q, Kitsis RN. Fundamental Mechanisms of Regulated Cell Death and Implications for Heart Disease. Physiol Rev. 2019; 99:1765–817. 10.1152/physrev.00022.2018 31364924 PMC6890986

[17] She Y. Preliminary study on pyroptosis involved in cerebral ischemia-reperfusion injury. Chin J Pathophysiol. 2019; 35:1379–86.

[18] Ye B, Chen X, Dai S, Han J, Liang X, Lin S, Cai X, Huang Z, Huang W. Emodin alleviates myocardial ischemia/reperfusion injury by inhibiting gasdermin D-mediated pyroptosis in cardiomyocytes. Drug Des Devel Ther. 2019; 13:975–90. 10.2147/DDDT.S195412 30988600 PMC6438141

[19] Ding S, Liu D, Wang L, Wang G, Zhu Y. Inhibiting MicroRNA-29a Protects Myocardial Ischemia-Reperfusion Injury by Targeting SIRT1 and Suppressing Oxidative Stress and NLRP3-Mediated Pyroptosis Pathway. J Pharmacol Exp Ther. 2020; 372:128–35. 10.1124/jpet.119.256982 31481517

